# Long term nitrogen deficiency alters expression of miRNAs and alters nitrogen metabolism and root architecture in Indian dwarf wheat (*Triticum sphaerococcum* Perc.) genotypes

**DOI:** 10.1038/s41598-023-31278-4

**Published:** 2023-03-27

**Authors:** Samrat Das, Dalveer Singh, Hari S. Meena, Shailendra K. Jha, Jyoti Kumari, Viswanathan Chinnusamy, Lekshmy Sathee

**Affiliations:** 1 Division of Plant Physiology, ICAR-IARI, New Delhi, India; 2 Division of Genetics, ICAR-IARI, New Delhi, India; 3 Division of Germplasm Evaluation, ICAR-NBPGR, New Delhi, India

**Keywords:** Natural variation in plants, Plant physiology, Plant stress responses, Plant sciences

## Abstract

The important roles of plant microRNAs (miRNAs) in adaptation to nitrogen (N) deficiency in different crop species especially cereals (rice, wheat, maize) have been under discussion since last decade with little focus on potential wild relatives and landraces. Indian dwarf wheat (*Triticum sphaerococcum* Percival) is an important landrace native to the Indian subcontinent. Several unique features, especially high protein content and resistance to drought and yellow rust, make it a very potent landrace for breeding. Our aim in this study is to identify the contrasting Indian dwarf wheat genotypes based on nitrogen use efficiency (NUE) and nitrogen deficiency tolerance (NDT) traits and the associated miRNAs differentially expressed under N deficiency in selected genotypes. Eleven Indian dwarf wheat genotypes and a high NUE bread wheat genotype (for comparison) were evaluated for NUE under control and N deficit field conditions. Based on NUE, selected genotypes were further evaluated under hydroponics and miRNome was compared by miRNAseq under control and N deficit conditions. Among the identified, differentially expressed miRNAs in control and N starved seedlings, the target gene functions were associated with N metabolism, root development, secondary metabolism and cell-cycle associated pathways. The key findings on miRNA expression, changes in root architecture, root auxin abundance and changes in N metabolism reveal new information on the N deficiency response of Indian dwarf wheat and targets for genetic improvement of NUE.

## Introduction

Nitrogen (N) is one of the essential nutrients required for crop growth and improved yield^1^. It is a crucial component of macromolecules like nucleic acids, proteins and secondary metabolites. Almost half of the applied N is lost from the soil for various reasons, including leaching, surface runoff, volatilization, denitrification etc. Hence farmers tend to apply more N fertilizers to the crops to mitigate the loss, resulting in environmental pollution, eutrophication, greenhouse gas emission. Besides, it is also cost-ineffective as it requires massive fossil fuels to manufacture synthetic fertilizers. Improving N use efficiency (NUE) is, therefore, the need of the hour^2^. Nitrogen use efficiency signifies grain dry matter per unit of N available from different sources^3^. It has been estimated that cereal NUE is only around 33% on average^4^, indicating excessive loss of N fertilizers, as discussed previously. Significant genotypic variations have been observed in terms of NUE in cereal crops earlier^5–7^. Hence the study of NUE and its related traits could be necessary selection criteria for better performing genotypes under low N and control conditions.

Nitrogen deficiency severely affects plants, such as leaf chlorosis, stunted growth, reduction in plant height, leaf area, tiller numbers, number of spikelets, grain biomass and final yield. Relative comparisons were made previously on N deficiency tolerance (NDT) traits (shoot and root biomass, plant height, root length and chlorophyll content) under low N and control conditions and these traits were considered as selection criteria for better adaptive genotypes in rice.^8,9^ analysed N assimilation enzymes such as nitrate reductase (NR), glutamine synthetase (GS), and glutamate dehydrogenase (GDH) activities which are good indicative of N metabolism as well as NDT. Besides, it was observed that auxin, which promotes root growth, showed a higher concentration near root meristems under N stress^10^. Therefore, the study of auxin concerning NDT is also imperative.

Among cereals, wheat is an important crop not just for its large-scale production and area but also for its nutritional value as a food grain. The total worldwide projected wheat production during 2022–2023 is 773.43 million tonnes in an area of 221.11 million hectares^11^. In India, the estimated production of wheat during the year 2020 was 107.59 million tonnes^12^. Since green revolution the yield potential of wheat has been increased, but it is not enough to feed the growing population in future. Wild wheat species possess potential traits which can be introgressed to modern cultivars for genetic improvement. Several promising results have been observed such as tolerance to drought stress^31^, better acquisition and utilization of nutrients^15^ etc. Indian dwarf wheat (*T. sphaerococcum* Perc.), commonly known as shot wheat, is a landrace of wheat cultivated in the Indian subcontinent since ancient times^16^. Moreover, it has also shown better N uptake capacity^17^. MicroRNAs (miRNAs) are a class of small, non-coding, approximately 22 nucleotide RNAs with regulatory roles in plants and animals^18^. Detailed information regarding N-responsive miRNAs and their responses to N deficiency in plants has been studied in maize and rice^19,20^. Researchers have also studied N-responsive miRNAs in bread wheat and durum wheat^21–25^. Several miRNAs associated with N deficiency response and NUE are reported in crop plants^26,27^. One of the N-regulated conserved miRNA, miR169a, targets transcription factor *NFYA* and overexpression of miR169a reduces N uptake due to suppression of nitrate transporters *NPF6.3* and *NRT2.1*^28^. Another miRNA, miR164 negatively regulates lateral root initiation through repression of its target *NAC1*^29^. miR399^30^ is found to be upregulated in maize^20^ and downregulated in *Arabidopsis*^31^ roots under N deficiency. The information on N regulated changes in miRNome of Indian dwarf wheat is highly limited. In this study, we identified Indian dwarf wheat genotypes with contrasting NUE and NDT traits, and through miRNAseq, miRNAs associated with N response were delineated. The results suggest an effective and coordinated signal transduction network involving miRNAs and their target candidate genes imparting adaptation to N deficiency.

## Materials and methods

All methods were performed in accordance with the relevant institutional, national, and international guidelines and legislation. The following traits of the studied genotypes were compared with a bread wheat genotype, BT-Schomburgk (BTS), as our previous field screening showed BTS as N responsive. Three experiments were performed, which include (1) preliminary field screening of 11 Indian dwarf wheat genotypes (S1–S11) and one bread wheat genotype (BTS) under N-sufficient (N+) and N-deficient (N−) conditions, (2) hydroponic evaluation of 3 genotypes identified from field screening and (3) miRNAseq analysis of 2 genotypes (Indian dwarf wheat and BTS) identified based on NUE, anthocyanin content and root traits from hydroponic evaluation.

### Field evaluation of genotypes

Twelve wheat genotypes (Supplementary Table 1) were grown under two N treatments in the field during *rabi* seasons (2018 and 2019) with the recommended application of 120 kg ha^−1^ N fertilizer in Urea form (N120 or N+) and without the application of N fertilizer (N0 or N-) at Division of Plant Physiology, ICAR-IARI, New Delhi (Supplementary Fig. 1A). The experiment was laid out in randomised block design. In N-field, the average native soil N content was 175 kg ha^−1^. The N-sufficient (N120) field received the recommended amount of nitrogen fertiliser in three separate applications: 50% N after field preparation, 25% N during the early vegetative stage, and 25% N during the late vegetative stage. Recommended dose of P and K fertilizers (60 kg ha^−1^) were applied as Single Super Phosphate and as Muriate of Potash) respectively as basal dose in the N120 and N0 fields. Prior to sowing the crop in the second season, the average soil N concentration in the N120 & N0 fields was 190 kg ha^−1^ and 156 kg ha^−1^, respectively. Soil P^H^ was approximately 6.9 in N0 and 6.95 in N120 fields while the electrical conductivity of soil solution was 3.95 dSm^−1^ N0 and 4.25 in N120 fields prior to planting. Before sowing, seeds of the genotypes were surface sterilized with 0.1% mercuric chloride (HgCl_2_) for 5 min and washed 5–6 times with double distilled water to remove the traces of HgCl_2_.

### Estimation of leaf area and biomass

The leaf area of plants was measured at vegetative stage (Zadok’s scale 28–29) approximately 40 days after sowing (DAS) in three replications per treatment for each variable using a Leaf area meter (LiCOR 3100, Lincoln, Nebraska, USA). Each replication consisted of three randomly selected samples. Total biomass was measured both at the vegetative stage (at 40 DAS) and at harvest in three replications per treatment in similar manner. Dry weight (DW) was measured after oven-drying the samples at 60 °C till constant weight was achieved.

#### Estimation of photosynthetic pigments and anthocyanin

The pigment content of plants was measured at vegetative stage (Zadok’s scale 28–29) approximately 40 days after sowing (DAS) in three replications per treatment for each variable, each replication consisted of samples poled from three randomly selected plants. Chlorophyll and carotenoid contents were measured following DMSO method^32^. The uppermost fully expanded fresh leaves were selected and cut into small pieces, excluding the midrib. These pieces were mixed up, and 25 mg samples were taken and put into test tubes containing 5 ml of dimethyl sulphoxide (DMSO). The test tubes were kept in an oven at 65 °C for 4 h under the dark condition to facilitate the extraction of chlorophyll pigments into the solution. Using a UV–visible spectrophotometer, the absorbance was measured at 470, 645 and 663 nm (Model Specord Bio-200, Analytik Jena, Germany). Total chlorophyll and carotenoid content were calculated according to^33^ and expressed as mg g^−1^ DW.

Anthocyanin content was measured using the protocol as described by^34^. Plant leaf sheath samples were collected freshly from the uppermost second node of the stem part. Plant material weighing 20 mg was taken from each sample and put into test tubes containing a 3 ml solution of 45% methanol and 5% acetic acid. It was kept overnight at ambient temperature and covered with aluminium foil to facilitate anthocyanin extraction into the solution. The absorbance was measured at 530 nm using a UV–visible spectrophotometer (Model Specord Bio-200, Analytik Jena, Germany), and anthocyanin content was expressed as mg g^−1^ FW.

### Analysis of yield parameters

At physiological maturity, plants were harvested, and estimates of biomass and grain yield per plant (g) were made. Different yield parameters, such as the total number of tillers per plant, the number of ears per plant, the number of spikelets per ear, length of ears (cm) etc., were also measured. Nine randomly chosen plants were sampled for all measurements. Plant height was measured after harvesting using a meter scale. The measurement was taken from the topmost part of the ear to the crown region of the shoot and expressed as cm plant^−1^.

### Estimation of total N content and NUE

Total N in the plant sample was measured following Kjeldahl's method^35^. After harvesting, both shoot and grain samples were finely ground for estimation. N content was expressed in percentage (%). N utilization efficiency (here denoted as NUE) was calculated from the biomass and N content of the respective grain samples and expressed as g grain g^−1^ N uptake.

### Hydroponic evaluation of selected genotypes

Three wheat genotypes were selected based on the field experiment. They were grown for 30 days in a plant growth chamber (Model PGW 36, Conviron, Winnipeg, Canada) in hydroponics in a controlled environment growth chamber at National Phytotron Facility, ICAR-Indian Agricultural Research Institute, New Delhi (Supplementary Fig. 1A-D). Seeds from each genotype were washed with double distilled water and surface sterilized with 0.1% HgCl_2_ for 5 min and rinsed thoroughly to remove the traces of HgCl_2_ 5–6 times with double distilled water, as discussed earlier. Then the seeds were kept for germination in Petri plates on germination paper. After 4–5 days, the germinated seedlings were transplanted to plastic trays containing 10 L of N-free Hoagland solution, each with two treatments having optimum N or N+ (7.5 mM NO_3_^−^) and low N or N− (0.05 mM NO_3_^−^) and kept inside growth chambers. The seedlings were supported on Styrofoam sheets (2″ thickness), and aquarium pumps were used to keep the solutions aerated. Each tray consisted of 3 genotypes in 5 replications, and each hill consisted of 3 wheat seedlings. There were three trays per treatment, and the arrangement of genotypes in different trays was randomised (Supplementary Fig. 1C-D); staggered batches of seedlings were raised to generate morpho-physiological observations.

#### Determination of growth parameters and pigment content

Leaf area and dry weight were measured by the method discussed above. Shoot fresh weight was measured immediately after harvest, while the fresh root weight was taken after removing excess water using blotting paper. Freshly harvested triplicate root samples of each genotype were scanned using a root scanner (Epson, Expression 11000XL, Graphic Art Model) with two replications per treatment and data analysed in Win-RHIZO, Regent Instruments Inc. software. Several root parameters were studied, such as total root length, total root surface area, average root diameter, total root volume, primary root length (diameter > 0.5 mm), lateral root length (diameter ≤ 0.5 mm), main root surface area (diameter > 0.5 mm), lateral root surface area (diameter ≤ 0.5 mm), main root volume (diameter > 0.5 mm) and lateral root volume (diameter ≤ 0.5 mm). Photosynthetic pigments (chlorophyll and carotenoid contents) and anthocyanin content were measured spectrophotometrically, as discussed earlier. The chlorophyll content index (CCI) was measured using an MC-100 chlorophyll concentration meter (Apogee Instruments Inc. Logan, UT, USA).

#### Estimation of nitrate reductase (NR) activity

NR activity of leaf and root samples were determined by the in-vitro assay^36^. For enzyme extraction, freshly harvested samples were homogenized in refrigerated 0.1 M phosphate buffer (pH 7.5) containing 5 mM EDTA and 5 mM cysteine. Extracts were centrifuged (sigma 3K30) at 10,000 rpm for 15 min at 4 °C^37^. The supernatant was collected and kept on ice. A reaction mixture containing 0.1 M phosphate buffer (pH 7.5), 0.1 M KNO3 and 10 mM NADH was prepared for assaying enzyme activity. Enzyme extract was added in test samples only to start the reaction, whereas in the blank sample, the extract was substituted with deionized water. To stop the reaction, 1 M zinc acetate was added with 75% ethanol, followed by centrifugation at 2000 rpm for 5 min at room temperature. The supernatant was collected, and 1% (w/v) sulfanilamide solution and 0.02% (w/v) *N*-(1-naphthyl) ethylene diamine solution were added to it^38^. After 20 min of incubation, absorbance was measured at 540 nm using UV–a visible spectrophotometer (Specord Bio-200, Analytik Jena, Germany). The enzyme activity was expressed as μmol nitrite formed mg^−1^ protein h^−1^.

#### Estimation of GS, GOGAT and GDH activity

GS (Glutamine synthetase), GOGAT (Glutamine oxoglutarate aminotransferase) and GDH (Glutamate dehydrogenase) activity of leaf and root samples were determined following^39^. Extraction was done in Tris–HCl buffer containing 100 mM Tris–HCl, 100 mM sucrose, 10 mM EDTA and 10 mM MgCl_2_. Extracts were centrifuged (sigma 3K30) at 5000*g* for 10 min at 4 °C. The supernatant was collected and re-centrifuged at 12,000*g* for 15 min at 4 °C. The supernatant obtained after the second round of centrifugation was used to assay GS and GOGAT. The pellet was dissolved in 50 mM phosphate buffer (pH 7.5) containing 2.14 g/100 ml sucrose and was used to assay GDH.

A reaction mixture containing 75 mM Tris buffer, 50 mM MgSO4, 5 mM cysteine, 125 mM α-glutamate, 5 mM ATP, and 10 mM hydroxylamine was prepared for assaying GS activity. Enzyme extract was added in test samples only to start the reaction, whereas, in the blank sample, the extract is substituted with deionized water. The mixtures were incubated for 30 min at 37 °C. To stop the reaction, 0.5 ml FeCl_3_ reagent was added, followed by centrifugation at 1500–2000*g* for 10 min. Absorbance was measured using a UV–visible spectrophotometer (Specord Bio-200, Analytik Jena, Germany). The activity of GS was expressed as µmol γ-glutamyl hydroxamate formed g^−1^ protein h^−1^.

To assay GOGAT activity, a reaction mixture containing 75 mM Tris–HCl, 10 mM α-ketoglutaric acid, 40 mM L-glutamine, and GDH activity containing 75 mM phosphate buffer, 20 mM α-ketoglutaric acid and 300 mM NH_4_Cl were prepared. The rest of the steps were similar to both. Enzyme extract was added in test samples only to start the reaction, whereas, in the blank sample, the extract was substituted with deionized water. 1.5 mM NADH was added to the reaction mixture before recording the absorbance at 340 nm for 60 s. The activity of GOGAT and GDH were expressed as µmol NADH oxidized g^−1^ protein h^−1^.

#### Estimation of soluble protein content

The soluble protein content of tissue extracts was measured by Bradford’s method^40^ to express enzyme activities in terms of the unit amount of protein. Bradford reagent (Genetix) was added to the plant extract prepared earlier, followed by vortexing. After 2 min of incubation, absorbance was measured at 595 nm using UV–a visible spectrophotometer (Specord Bio-200, Analytik Jena, Germany).

#### Estimation of tissue nitrate content

The tissue nitrate content of the leaf and root samples was measured following the hydrazine sulphate reduction method^41^. Oven-dried samples were finely ground for estimation. An equivalent amount of sample and nitrate-free charcoal were taken in a conical flask containing 15 ml of double-distilled water and mixed thoroughly. It was then boiled for 3–4 min and filtered through Whatman filter paper-42 followed by re-extracted and refiltration and volume made up to 50 ml. After 20–30 min of incubation, absorbance was recorded at 540 nm using UV–a visible spectrophotometer (Specord Bio-200, Analytik Jena, Germany).

#### Estimation of IAA content and IAA localization

Natural auxin indole-3-acetic acid (IAA) content was estimated using the method described by^42,43^. Fresh root and shoot samples were ground in liquid N to form a fine powder. 2.5 ml g^−1^ FW of 100% methanol was added and kept overnight at 4 °C in dark conditions. It was centrifuged afterwards at 16,000*g* for 10 min at 4 °C, and the supernatant was transferred to a new fresh tube. 2 ml of 100% methanol was added and kept in the dark condition at 4 °C for 1–2 h, followed by centrifugation under the same condition. The supernatant volume was reduced to 1/10th in a speed vacuum. 1 ml of HPLC water was added, and pH was adjusted to > 9.0 with 1 M KOH using pH strips. 100% ethyl acetate was added to double the volume and centrifuged at 16,000 g for 10 min at 4 °C. The lower aqueous phase was taken in a new tube, and pH was adjusted further to < 3.0 with concentrated acetic acid using pH strips. An equal volume of 100% ethyl acetate was added and centrifuged at 16,000* g* for 2 min at 4 °C. The upper organic phase was taken and was entirely dried in a speed vac. Finally, it was dissolved in 60:40 methanol (300 μl) and filtered using 0.45 μm PVDF syringe filters. For mobile phase preparation, solvents A and B were prepared using 90% methanol with 0.3% acetic acid and 10% methanol with 0.3% acetic acid, respectively, and were filtered through a 0.45 μm PVDF membrane filter degassed by a vacuum pump. The HPLC system estimated IAA concentration in the reverse phase C_18_ column (Apollo C_18_, five μm, Alltech, USA) (Agilent Technologies 1200 Infinity). The samples were run under standardized conditions, and peaks were detected in fluorescence detector (FLD) with an excitation wavelength of 280 and emission wavelength of 360 nm, the retention time varied from 15.5 TO 16.5 min. Standard solutions of IAA were used for the identification of peaks. The concentration of IAA was quantified from the calibration curves of the standards and expressed in μg g^−1^ FW.

The colourimetric assay of IAA was done using the Salkowski reagent. Salkowski reagent was prepared by mixing 0.5 M ferric chloride (FeCl_3_) and 35% perchloric acid (HClO_4_). 1–2 ml Salkowski reagent was put in watch glasses. Plant root tips were cut and dipped into it for 1 h under complete darkness by covering with black cloth. Whole plant roots were also submerged separately. The pink colour appeared where IAA concentration is more in root and was observed under EVOS XL microscope (AMG, Bothell, WA).

### Extraction of miRNA from wheat genotypes and miRNAseq analysis

miRNAs were extracted from the leaf and root tissues of selected genotypes (S3 and BTS) based on hydroponic evaluations with the mirVana miRNA isolation kit (AM1560, Life Technologies, Carlsbad, CA) according to the manufacturer's protocol. RNA was quantified with a Nanodrop ND2000 spectrophotometer (Thermo Fisher Scientific, Waltham, MA). For miRNA sequencing (NGS), samples were sent to Nucleome Informatics Private Ltd, Hyderabad, India. Small RNA was extracted at three-time points, the 7th day, 14th day and 21st days after treatment with control (N+) or N deficiency (N−) treatments from leaf and root tissues separately from genotypes S3 and BTS. The quality and quantity of total RNA isolated from each sample were further analysed by Bioanalyzer 2100 (Agilent Technologies). The RNA from six samples were used for small RNA library preparation using a Small RNA Sample Preparation Kit (Illumina Technologies) following the manufacturer’s instructions. The small RNA libraries were sequenced using a Hiseq Illumina 1.5. The sequence FASTQ files were analysed by FastQC ver 3 and Fastx-toolkit, ver 0.0.13. The raw sequences were then trimmed to omit adaptor/primer contamination and poly(A) tail with miR Deep adaptor filter. The unique reads having 17–23 nucleotide long sequences were retained for mapping. The reads were screened against non-coding RNA sequences found in the wheat genome database (https://plants.ensembl.org/Triticum_aestivum/Info/Index). The conserved miRNA sequences were recognized using RNA central database (https://rnacentral.org/) and mapped employing the Bowtie alignment tool^44^. A maximum of two mismatches was allowed for analysis. EdgeR (R package, version 3.8.3) software was utilized to identify the differential gene expression for miRNAs.

### Statistical analysis

Two-way analysis of variance (ANOVA) was carried out in GraphPad Prism version 8 (La Jolla, California, USA) with variety, N treatments as treatment effects to compute adjusted P values and significance levels. Mean separation was done using Sidak's multiple comparisons test following one-way ANOVA. Graphs and heatmaps were prepared using GraphPad Prism version 8 (La Jolla, California, USA). For yield data recorded in seasons 2018–2019 and 2019–2020, data was analysed in OPSTAT to compute statistical significance of cropping season. Statistical significance of cropping season on genotype wise yield was compared by multiple t test in GraphPad Prism version 8.

## Results

### Effect of N deficiency on physiological and yield parameters

Leaf area (cm^2^) (at 40 DAS), biomass accumulation (g) (at 40 DAS) and total biomass accumulation (g) (at harvest) of wheat genotypes were significantly different concerning N level, variety and their interaction (Supplementary Fig. 2). Sidak's multiple comparisons test compared the varietal variation among N+ and N− treatments. In N− treatment, the varietal mean of leaf area (at 40 DAS) was significantly lower in genotypes S3, S5, S7 and S9, but not significantly different among treatments in BTS, S1, S2 etc. Under N-treatment, there was approximately a 47% reduction in leaf area compared to N+ treatment (Supplementary Fig. 2a). Regarding the biomass accumulation (at 40 DAS), varietal means were significantly lower in genotypes like S2, S3, S4, S5, S9, S11, whereas varietal means were not significantly different in S1, S6 etc. (Supplementary Fig. 2b). Regarding the total biomass accumulation (at harvest), varietal means were substantially lower in genotypes like S4, S5, S6, S8, S9, S10, whereas varietal means were not significantly different in BTS, S1, S2, S3, S17 etc. (Supplementary Fig. 2c). Plant height (cm) and yield parameters such as mean values of tiller numbers per plant, number of ears per plant, spikelets per ear, ear length (cm) of wheat genotypes were significantly different in N level, variety and their interaction except for the treatment means of tiller number (Supplementary Figs. 3 and 4). Sidak's multiple comparisons test showed that varietal means of plant height were significantly lower in all the genotypes. Plant height was reduced considerably by N deficiency (Supplementary Fig. 3a). Genotypic mean values of N− treatment of all yield parameters were significantly lower than that of corresponding N+ treatment in all varieties, as depicted by Sidak's multiple comparisons test (Supplementary Figs. 3b, c and 4). The grain yield per plant was evaluated in cropping seasons (Supplementary Table 2), there was significant effect of N treatment and genotypes, while the year effect was significant among year 1 and 2 N+ treatment in S2, S7 and S8.

### Effect of N deficiency on pigments and NUE

**Figure 1:**
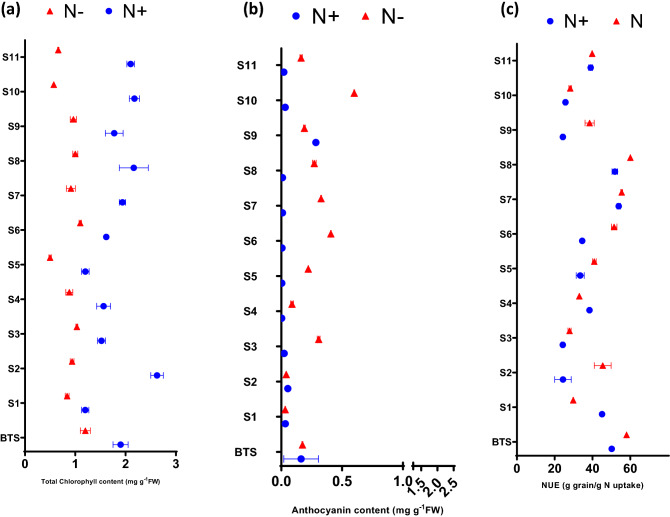
Effect of nitrogen deficient (no applied N: N−) and nitrogen sufficient (120 kg ha^−1^ applied N: N+) field conditions on (**a**) total chlorophyll content, (**b**) anthocyanin content and (**c**) nitrogen utilization efficiency (NUE) of wheat genotypes.

Significant differences were observed in mean values of total chlorophyll content, total carotenoid content and anthocyanin content with regard to N level, variety and their interaction except for treatment differences in carotenoid content (Fig. 1). There was a general reduction in total chlorophyll (50%) content in N− treatment. There was a significant increase in total carotenoid in S2, S6, S7 (data not shown). Total chlorophyll content decreased in Indian dwarf wheat compared to bread wheat; however, some good contrasts were present in the evaluated germplasm. For example, S1 (30%) and S3 (32%) performed exceptionally well under N deficient conditions (Fig. 1a). Wheat genotypes accumulated more anthocyanin in response to N deficiency. Anthocyanin content increased manifold in Indian dwarf wheat in comparison to bread wheat. There were some good contrasts in N starvation response (NSR) present in the evaluated germplasm; for example, S4 (24%), S5 (50%), S6 (67%) accumulated a significantly higher amount of anthocyanin content under N deficient condition (Fig. 1b). Significant differences were found in mean values of NUE with regard to N level, variety and their interaction. Sidak's multiple comparisons tests showed that the mean NUE of N− treatment was significantly different from N+ treatment in the varieties, S1, S2, S5, S9 and were not significant in BTS, S3, S4 etc. (Fig. 1c). Based on NUE and NSR responses, genotypes selected for further detailed studies were BTS (high NUE), S2 (high NUE), S3 (low NUE but not affected by N level).

### Effect of N deficiency on seedling morpho-physiological traits in hydroponic culture

**Figure 2:**
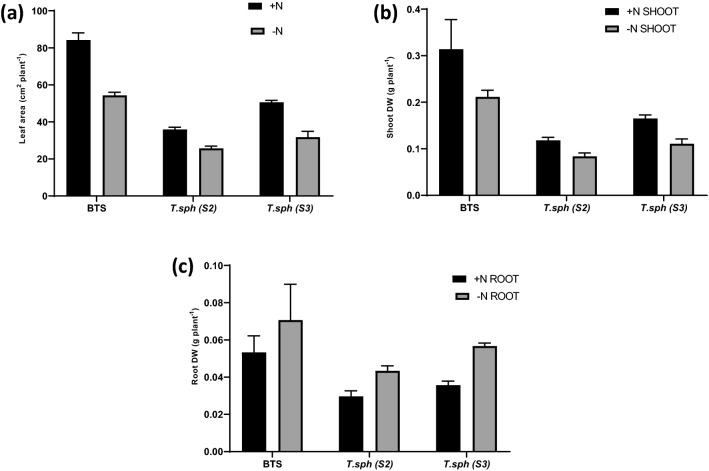
Effect of nitrogen deficient (0.05 mM nitrate, N−) and nitrogen sufficient (7.5 mM nitrate, N+) conditions on leaf area (**a**), shoot dry weight (**b**) and root dry weight (**c**) of thirty days old seedlings of wheat genotypes grown under hydroponic culture.

Mean values of shoot dry weight, root dry weight and leaf area were significantly different with regard to N level, variety and their interaction (Fig. 2). Genotypic mean values of leaf area and shoot biomass of N− treatment of all the genotypes were lower than corresponding N+ treatment. Varietal leaf area means were significantly different in BTS and S3, as indicated by Sidak's multiple comparisons test (Fig. 2a). Shoot and root dry weight data of the seedlings were also recorded. N deficiency leads to yellowing of lower leaves, reduces leaf area by 31%, and shoots dry weight by 25% in wheat seedlings. Varietal means of shoot dry weight was significantly different in BTS and S3, as depicted by Sidak's multiple comparisons test (Fig. 2b). Root dry weight showed an increase of 2.6% in the N deficit condition (Fig. 2c).

**Figure 3:**
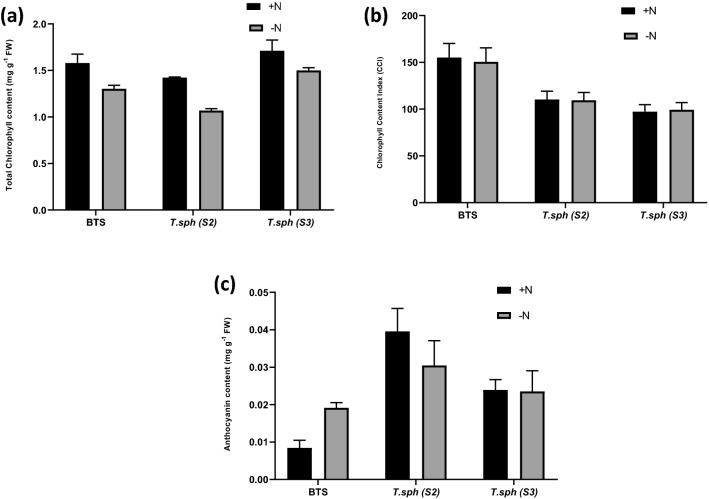
Effect of nitrogen deficient (0.05 mM nitrate, N−) and nitrogen sufficient (7.5 mM nitrate, N+) conditions on total chlorophyll content (**a**) CCI (**b**) and anthocyanin content (**c**) of thirty days old seedlings of wheat genotypes grown under hydroponic culture.

The differences in total chlorophyll, CCI values and anthocyanin content were significant at different N treatments and genotypes and the interaction of variety and treatments except for the genotypic effects in anthocyanin content. The reduction in chlorophyll content was lowest in BTS. Indian dwarf wheat showed a considerable reduction in chlorophyll retention in N− condition (Fig. 3).

**Figure 4:**
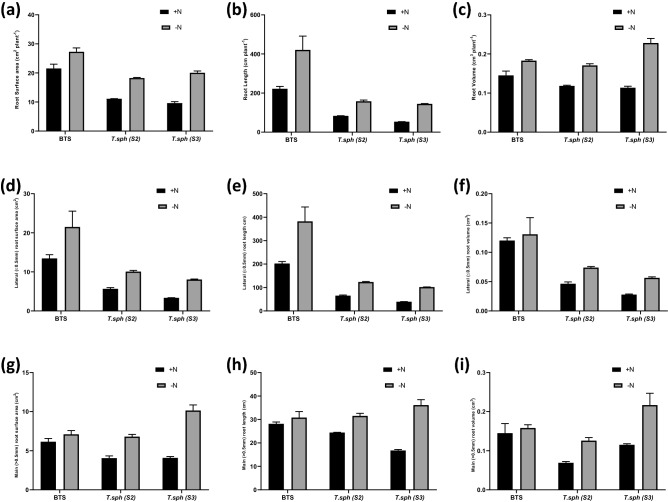
Effect of nitrogen deficient (0.05 mM nitrate, N−) and nitrogen sufficient (7.5 mM nitrate, N+) conditions on surface area (**a**, **d**, **g**), root length (**b**, **e**, **h**) and root volume (**c**, **f**, **i**) of total, lateral and main roots of thirty days old seedlings of wheat genotypes grown under hydroponic culture.

**Figure 5:**
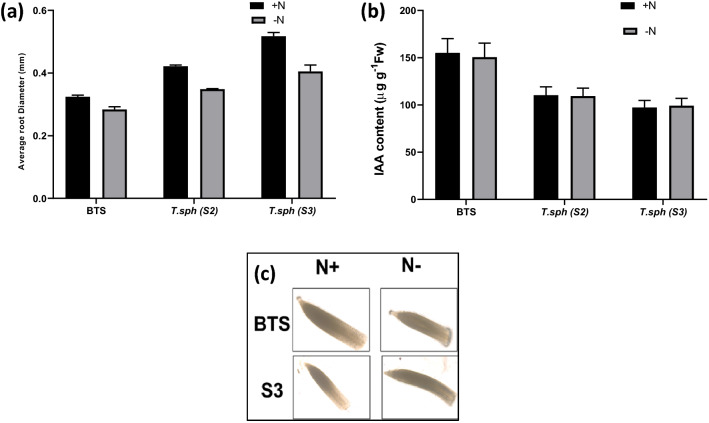
Effect of nitrogen deficient (0.05 mM nitrate, N−) and nitrogen sufficient (7.5 mM nitrate, N+) conditions on average root diameter (**a**), IAA content (**b**) and IAA localization in root tips (**c**) of thirty days old seedlings of wheat genotypes grown under hydroponic culture.

Significant differences were observed in root traits like average root diameter, total root length, total root volume, total root surface area, length of the main root (diameter > 0.5 mm), length of lateral root (diameter ≤ 0.5 mm), the volume of the main root, volume of the lateral root, the surface area of the main root and surface area of lateral root with N treatments and varieties (Figs. 4, 5a). Irrespective of genotype, the N− condition significantly improved the root growth, root surface area and the number of fine root hairs in wheat (Supplementary Fig. 5). Genotypic variations in root traits were also significant. Root proliferation also has a significant correlation with nitrate uptake kinetics. An increase in root growth is an important adaptation to NSR and was most prominent in S3 depicted as robust root system which is evident visually in (Supplementary Fig. 5). The treatment effects on average root diameter were significantly lowered by N deficiency (Fig. 5a). Lateral root length and lateral root volume were found to be important parameters affected by N availability. Genotype BTS showed an increase in total and lateral root length (88 and 89%) under N deficiency (Fig. 4).

### Effect of N deficiency on root IAA content and localization

The IAA content and localization in root tips were studied in wheat seedlings grown with either N+ or N− treatment (Fig. 5b, c). The highest root IAA content was found in BTS. In BTS, S2 and S3, IAA content remained unchanged by treatments (Fig. 5b). To further understand IAA content and correlate with NSR associated root growth, IAA localisation in root tips was also analysed. In root tips of N deficient plants of BTS and S3, IAA localisation was more prominent, as depicted in the photomicrographs. The IAA abundance of BTS is similar under both treatments, whereas S3 shows darker colouration in N deficiency treatment (Fig. 5c).

### Effect of N deficiency on leaf and root N metabolism of wheat seedlings

**Figure 6:**
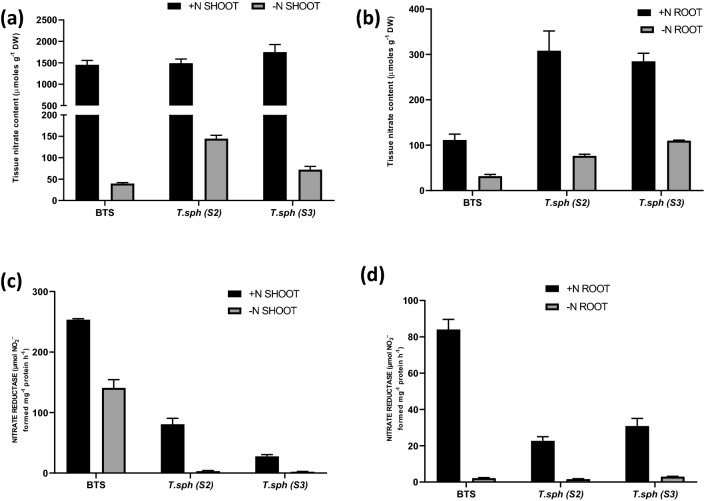
Effect of nitrogen deficient (0.05 mM nitrate, N−) and nitrogen sufficient (7.5 mM nitrate, N+) conditions on tissue nitrate content (**a**, **b**) and nitrate reductase (NR) activity (**c**, **d**) of thirty days old seedlings of wheat genotypes grown under hydroponic culture.

**Figure 7:**
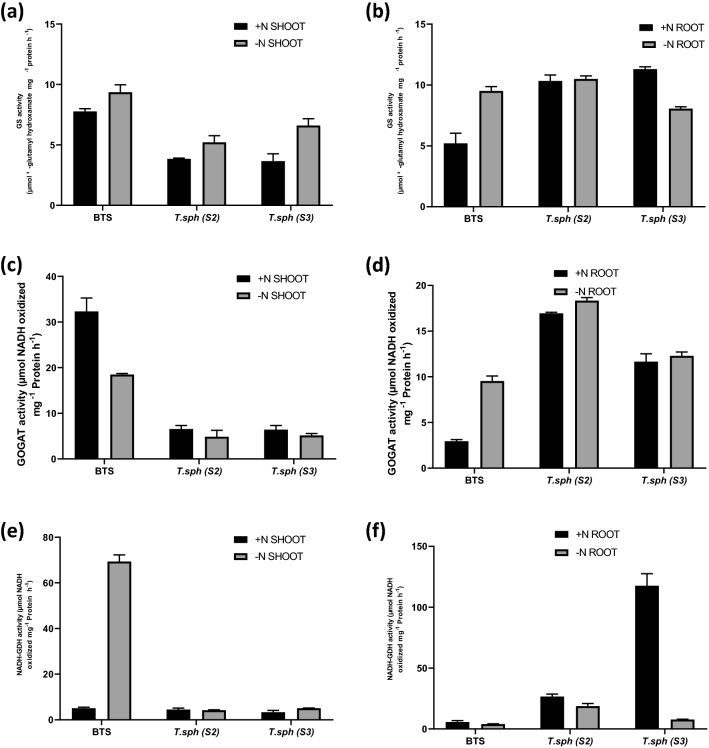
Effect of nitrogen deficient (0.05 mM nitrate, N−) and nitrogen sufficient (7.5 mM nitrate, N+) conditions on activities of nitrogen assimilatory enzymes glutamine synthetase (GS) (**a**, **b**), glutamate synthase (GOGAT) (**c**, **d**) and glutamate dehydrogenase (GDH) (**e**, **f**) in thirty days old seedlings of wheat genotypes grown under hydroponic culture.

Shoot nitrate content was significantly reduced by N deficiency, approximately 95% decline in all the genotypes. However, the highest tissue nitrate content in N+ conditions was found in S3. Under N− condition, S2 and S3 showed higher root nitrate content than BTS (Fig. 6a,b). In seedling leaves and roots, NR activity was highest in the N+ condition. In the N+ condition, BTS recorded the highest leaf NR activity, and the lowest was recorded in S3. NR activity in roots was fourfold lower than that of leaves (Fig. 6c,d). Total GS activity showed significant up-regulation under N deficit in leaves and roots. Varietal differences were also significant; the highest activity among seedling leaf and root observations was in BTS. Genotypes S2 and S3 showed low leaf GS activity and high root GS activity (Fig. 7a,b). GOGAT activity was highest in leaves in comparison to roots, however, in N deficiency subdued the activity (by 55%), in leaves, the reverse was true (increase by 2.5%). In leaves, N deficiency tremendously increased the GOGAT activity in BTS. The highest root GOGAT activity was found in genotypes S2 and S3, which showed an increment of 8% and 6%, respectively, under the N− condition (Fig. 7c,d). GDH activity was highest in roots compared to leaves; however, in leaves, N deficiency subdued the activity (by 55%), in roots, the reverse was true (increase by 6%). Under N deficiency, BTS showed the highest leaf GDH activity (13.6 fold higher than N+). The highest root GDH activity was found in, S3 which decreased by 93%, under N− condition (Fig. 7e,f).

### miRNAseq analysis and identification of low N responsive miRNAs and qPCR validation of selected transcripts

**Figure 8:**
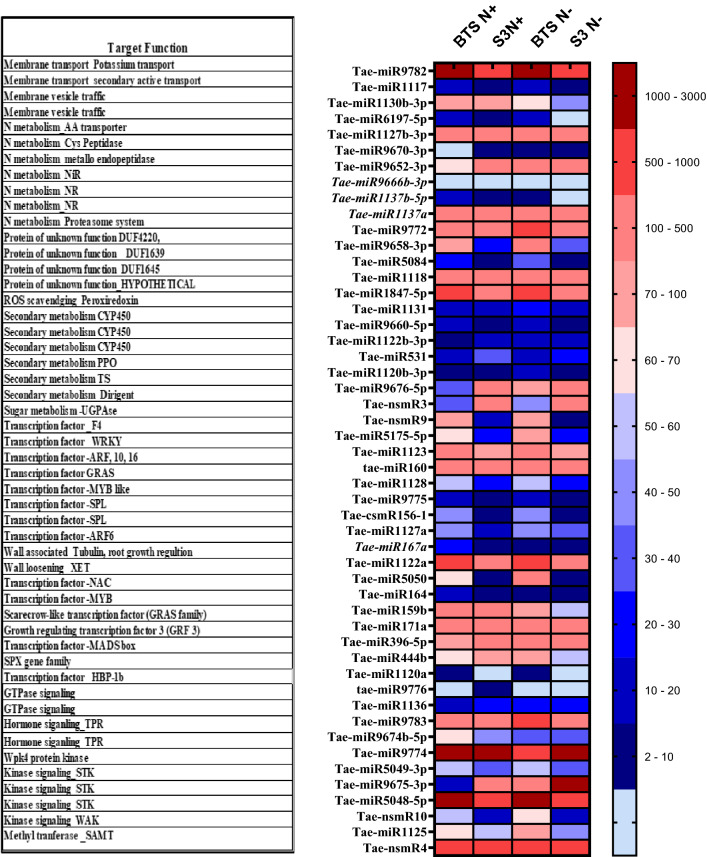
Expression heatmap of differentially expressed miRNA transcripts and the functions of respective target genes from miRNAseq analysis of genotypes BT-Schomburgk (BTS), and* T. sph* (S3) subjected to control (N+) or N deficient (N−) conditions.

Based on miRNAseq analysis, approximately 50 miRNAs were differentially regulated in response to N deficiency in two genotypes (BTS and S3). The expression details of the miRNAs most significantly (in terms of fold values) affected by N deficiency is presented in Fig. 8. With regard to the most differentially regulated miRNAs, the respective target genes belonged to the following four classes mainly Kinases (Ser-Thr, Wall associated), N metabolism (Purine metabolism, AA transporter, Cys Peptidase, Metallo endopeptidase, NiR, NR, Proteolysis, Proteasome system), Secondary metabolism (CYP450, PPO, Terpene synthase), Dirigent protein (Lignin biosynthesis), Transcription factor- F4, WRKY, ARF, GRAS, MYB like SPL, NAC. Differential expression of N metabolism-related miRNAs suggests reprogramming N assimilation and remobilisation machinery (Cys Peptidase, Metallo endopeptidase, NiR, NR, Proteolysis, Proteasome system). Similarly, secondary metabolism-related miRNAs expression (targeting terpene synthase, polyphenol oxidase and lignin biosynthesis) also points out the change in NSR in terms of secondary metabolites and anthocyanins. miRNAs associated with N regulated Root growth, for example, ARF6, ARF10, ARF16, GRAS family TF, Wall loosening related genes, were reprogrammed- altered RSA under N deficiency. miRNAs targeting Kinases such as Ser-Thr Kinases, Wall associated PK, WPK, hormone signalling (Tetratricopeptide-like helical domain superfamily) were also differentially regulated- N Signal perception and signaling.

## Discussion

It is estimated that a 1% increase in cereal NUE can compensate for the cost of N fertilizers, amounting to more than 200 million US dollars on a global scale^4^. Nitrogen use efficiency is a polygenic and complex trait, and its study requires a multi-disciplinary approach. miRNA mediated regulation of gene expression in response to abiotic stresses has already been well-studied in plants^45,46^. Our objectives in this study were to evaluate Indian dwarf wheat genotypes based on NUE and miRNAseq analysis was carried out to identify miRNAs related to NUE and N deficiency. The study shows that both high and low NUE genotypes identified based on NUE, anthocyanin content and root traits have a coordinated network of the miRNA-target module in which most of the targets show similarities in terms of their functions.

The previous studies have shown that N deficiency causes chlorosis of lower leaves and reduces leaf area and shoot growth in rice seedlings, with noticeable results in PB1 indicating that seedling stage screening in the field is necessary for NUE assessment^47^. The reduction in photosynthesis, remobilization of N to developing parts and anthocyanin pigmentation are some of the key responses of plants to low N^48,49^. In this study, varietal mean of all the growth parameters such as leaf area, biomass accumulation, plant height and other yield-related attributes were significantly reduced under N− condition. High NUE genotype BTS showed the lowest reduction. Anthocyanin accumulation was relatively higher in Indian dwarf wheat (21%) grown in the field under N starved condition with some good contrasts found in S4 (24%), S5 (50%), S6 (67%) genotypes. Significant reduction in chlorophyll a, chlorophyll b, total chlorophyll content, chlorophyll a/b ratio and carotenoid content were also observed in low NUE genotypes compared to high NUE under limited N treatment, suggesting that chlorophyll content is a good indicator of NUE. (Padhan et al.).

In simple terms, NUE is the ratio of plant biomass or grain yield to available N in soil and supplied N^50^. Molecular breeding and marker-assisted selections could show promising results with regard to NUE improvement^51^ but NUE being a complex trait, efficient screening methods are required to accomplish this^52^. Here we performed preliminary field screening and found variations in NUE among genotypes. Studies showed that increasing N fertilizers application will not increase the yield beyond optima but certainly increase grain N content^53^. Similar results were also obtained in our study, where a significant improvement in grain N content was observed under N sufficient conditions in the field, specifically in high NUE genotypes. Grain N content can be an essential NUE attribute^54^. Based on NUE, the following genotypes such as BTS (High NUE), S2 (high NUE), S3 (low NUE but not affected by N level) were selected for further analysis. Screening for NUE related traits in plants is more rapid, effective, uniform and economically feasible under a hydroponic system than field screening^55^. Hence in this study, selected genotypes were further grown and analysed in hydroponics for change in growth, root traits, photosynthetic pigments and anthocyanin content, N assimilation enzymes activity, tissue nitrate content, soluble protein content, IAA content and localisation.

### NSR Adavi and Sathee

In general, N stress induces root proliferation to a certain extent in all genotypes for better forage of nutrients^56^. In this study, S3 depicted robust root growth in N− treatment with clear visual evidence. Significant differences were observed in root traits like total root length, total root volume, total root surface area, primary root length, lateral root length, main root volume, lateral root volume, main root surface area and lateral root surface area for N treatments and varieties. As discussed previously, anthocyanin accumulation is a significant aspect of NSR, and our findings align with this observation. It was reported earlier that lateral root growth is governed by auxin^57,58^. Auxin promotes lateral root initiation through pericycle cell division^59^. In BTS, S2 and S3, the effect of N treatment on root IAA content was minimal. While IAA localisation was more visually prominent in root tips of N deficient plants of S3, IAA abundance in BTS root tips was similar under both treatments.

N assimilation enzymes are essential for the adaptability and survival of plants^60,61^. NR activity is induced under low N supply and vice-versa^62,63^. GS and GOGAT activities also respond similarly^64,65^. Assimilatory GDH activity increased due to the application of external N^66^, whereas dissimilatory GDH activity showed the opposite result^67^. We found that N assimilatory enzyme activity was several times higher in shoots than in the roots, irrespective of treatments. Although low N induces NR activity to some extent, NR activity was found significantly lower in N− treatment than N+ , possibly due to the low availability of nitrate (substrate). We found the highest NR activity in BTS (high NUE) in leaves irrespective of treatments.

Similarly, GS activity increased almost twice under the N− condition. Overall, GS activity was high in low N, mainly due to higher cytosolic GS or GS1 activity^68^. Therefore, it can be concluded that secondary N assimilation in photorespiratory ammonia is higher under the N deficit condition by the activity of GS1. GOGAT activity was also higher in low N. There was a significant increase in GDH activity by N deficiency in leaves of wheat genotypes. We selected genotypes BTS and S3 for miRNAseq analysis based on NUE, anthocyanin content, and root traits.

Nutrient deprivation, especially essential nutrients, poses severe threats to plant growth. To cope with such problems, plants usually take various means in which the miRNA-target module plays a vital role. As discussed earlier, miRNAs mediate gene regulation for low N adaptability in plants. In this study, miRNAseq analysis showed around 100 miRNAs differentially regulated under N deficiency in BTS and S3, in which 50 miRNAs were most significantly (in terms of fold values) affected by N deficiency. The expression details revealed that the target gene functions of the miRNAs are as follows: fatty acid metabolism (enoyl isomerase, FAD, lipase), GTPase signaling, hormone signaling, serine/threonine-protein kinase (STK), wall-associated kinase (WAK), WPK, membrane transport (potassium transport, secondary active transport), membrane vesicle traffic, methyltransferase- SAMT, N metabolism- (purine metabolism, AA transporter, Cys peptidase, metalloendopeptidase, NiR, NR, proteolysis, proteasome system), DUF4220, DUF668, DUF1639, DUF1645, HYPOTHETICAL protein, ROS scavenging, peroxiredoxin, secondary metabolism CYP450, PPO, terpene synthase. Dirigent protein, sugar metabolism- UGPase, transcription factor- F4, WRKY, ARF, GRAS, MYB like SPL, Tubulin, XET etc. With regard to the most differentially regulated miRNAs, the respective target genes belonged to the following four functional classes, mainly as Kinases (Ser-Thr, wall-associated), N metabolism (purine metabolism, AA transporter, Cys peptidase, metalloendopeptidase, NiR, NR, proteolysis, proteasome system), secondary metabolism (CYP450, PPO, terpene synthase) and dirigent proteins (lignin biosynthesis), transcription factor- F4, WRKY, ARF, GRAS, MYB like SPL, NAC.

N deficit impacts all areas of plant function, including metabolism, resource allocation, growth, and development, as N is a major component of amino acids, nucleic acids, chlorophyll, ATP, coenzymes, and plant hormones^69^. The wall-associated receptor kinases (WAKs), are principally engaged in the regulation of plant cell wall functions and control cell growth, morphogenesis, and development Anderson. Deficit of N stimulated the expression of WAK125 (Os12g0478400) and WAK37 (Os04g0365100) in rice roots. It's interesting to note that WAK125 was already identified as an early glutamate-responsive gene^70,71^. The expression of Os09g0442100 and Os06g0142650, which encode RLK homologs, was similarly quickly promoted by N deficiency, in addition to WAK125 and WAK37^72^. Transcriptome study of ammonium-responsive genes in rice roots revealed the Os02g0120100 gene encoding ACT domain-containing protein kinase 1 (ACTPK1), a homolog of Arabidopsis serine/threonine/tyrosine kinase 46 (STY46), that can phosphorylate and inactivate ammonium transporter AMT1;2^73^. WPK4 is a wheat protein kinase that effectively phosphorylated the hinge 1 region of nitrate reductase and post translationally regulate NR activity^74^. In rice, roots the expression of os10g0576600, which encodes a tetratricopeptide repeat (TPR) protein was induced by N sufficient and N deficient conditions^72^.

In Arabidopsis, it has been demonstrated that elements of the ubiquitin-mediated proteolytic degradation system modify N responses. An E3 ubiquitin-protein ligase complex (CUL3-RBX1-BTB) controls the ubiquitination and subsequent proteasomal degradation of target proteins. In rice, nitrate transporter genes and N use efficiency are negatively regulated by the rice BT2 homolog (Os01g0908200)^75,76^. It's interesting to note that the RING-type E3 ubiquitin-protein ligase EL5-like genes BT2 and Os05g0360400 are N-sensitive genes^76^. Nutrient deficiency including that of N increases ROS production in Arabidopsis and rice^77^. In rice roots, N deficiency activated genes encoding peroxidase, peroxidase-like proteins (and Glutathione-S transferase. Similarly, the expression of oxidative stress responsive genes GolS1 (Os03g0316200) and GolS2 (Os07g0687900), which encode galactinol synthase, a crucial enzyme for the manufacture of raffinose family oligosaccharide^78^. These findings suggest the generation of ROS and redox signalling pathways are among the earliest events connected to N deficiency. In rice, WRKY33 (Os01g0826400) Os03g0609500 (LBD38), Os03g0445700 (LBD37), Os07g0589000 (LBD37), Os05g0114400 (ZOS5-02), Os11g0184900 (NAC5), Os07g0119300 (MYB), and Os03g0764600 (MYB) and CIPK14 are among the 34 N-sensitive genes^72^. The heterotrimeric guanine nucleotide binding protein (G-protein), has been suggested as a key modulator of N response in rice, wheat, and Arabidopsis^79^. TaNBP1 has been found to encode G proteins in wheat, which increase N uptake under N deficit conditions^80^. Similar to this, in rice, the dep1-1 allele of the G Protein enhances N uptake, N assimilation and harvest index under moderate N supply^81^. Gene ontology enrichment study of rice seedling roots has showed that N-deficiency enhanced expression of two jasmonic acid biosynthesis biosynthesis genes, Os08g0508800 and Os03g0738600, that encode chloroplastic lipoxygenase 7 and linoleate 9S-lipoxygenase 2, respectively^72^.

Like many other signalling-related genes, phosphate signalling repressor *SPX4* is involved in nitrate signalling^82^. Some of the targeting miRNAs of those genes in wheat include tae-miR1120a, tae-miR1120b-3p, tae-miR1120c- 5p, tae-miR1122b-3p, tae-miR1122c-3p, tae-miR1130a, tae-miR1130b-3p, tae-miR1137a, and tae-miR1137b-5p (data obtained through in silico analysis, not shown here). In the present study, miRNAseq analysis showed that tae-miR1137b-5p, tae-miR1137a, tae-miR1122b-3p, tae-miR1120b-3p were differentially expressed. Apart from SPX, other targets of these miRNAs were NUE-related traits such as tae-miR1137a and tae-miR1137b-5p target NR, tae-miR1122b-3p and tae-miR1120b-3p target genes associated with secondary metabolism. It has also been observed that tae-miR156, which is conserved functionally, regulates plant morphology, and its overexpression causes excessive tillering giving a bushy appearance and defective spikelets^83^. In this study, expression details demonstrated that tae-csmR156-1 was differentially expressed and conserved in nature; it might also regulate plant morphological changes^84^. reported an inverse relationship between miR160-*ARF16/17* and miR167-*ARF6/8* modules stimulating lateral root growth in *Arabidopsis* under N-starved conditions. The differential expression of miR160 and miR167a in the current study can thus be correlated with the changes in root growth under N deficiency^25^. Studied N responsive miRNAs in bread wheat, and miR159, miR399 and miR408 were reported to be differentially expressed^23^. Also found tamiR444a regulating plant tolerance to N stress. Another report showed that the NSR-regulated miR396 targets the Growth Regulating Factor (GRF) to regulate leaf development while miR171 targets SCARECROW-like (SCL) transcription factors and regulates root growth in *Arabidopsis*, maize and soybean^85^. Similarly, our results are at par, showing altered expression of miR159b, miR171a, miR396-5p, miR444b etc. We found an approximately twofold increase in miR171 expression in BTS under the N− condition. There are four classes of miRNAs based on their conservativeness^27^. The most conserved group includes miR164, miR172, miR398 and miR399, whereas, highly conserved are miR156, miR167, miR395, miR319, miR408 and miR2111; miR160, miR168, miR166 and miR397 belong to moderately conserved and less conserved group consists of miR158, miR159, miR169,miR170, miR171, miR528, miR390, miR396, miR394 and miR827 y^27^. Some of the unique miRNAs regulated under P deficiency such as miR437, miR447, miR771, miR775, miR778, miR830, miR837, miR896, miR1122, miR1125, miR1135, miR1136, miR1211, miR1222 and miR1507 target *PHO2*, *ARF6*, *ARF8* and *AP2* genes. Some N-regulated unique miRNAs include miR826, miR829, miR839, miR846, miR850 and miR863; target genes are *NLA, ARF6, ARF*8*, ARF*16*, ARF*18*, HAP2* and *AP*2. Our study found differential regulation of some unique miRNAs such as miR1125, miR1136 and miR1222 in N deficient wheat seedlings. *PHO2* encodes a ubiquitin-conjugating E2 enzyme 24, known to play a role in maintaining phosphate homeostasis. However, the differential expression of miRNAs targeting *PHO2* in the current study establishes the role of PHO2 under N deficiency. Differential expression analysis suggests that these discovered members of miRNAs are involved in reprogramming N assimilation and remobilisation machinery and developing secondary metabolites and anthocyanin in response to N deprivation. N regulated root growth and actions of different kinases (Ser-Thr kinases, wall-associated protein kinases etc.) for downstream signal transduction are also reprogrammed.

In conclusion, the wheat homologues of N-responsive miRNAs mediate NUE-related responses through downstream signalling pathways. miRNAseq analysis revealed the expression details of miRNAs involved in signal transduction in high NUE Indian dwarf wheat (*Triticum sphaerococcum* Perc.) genotypes identified under low N conditions. Our findings infer that the effective and coordinated signal transduction network involving the miRNAs targeting and reprogramming N metabolism, secondary metabolism, N signal perception and signalling helps to achieve high NUE. The miRNAS targeting key genes could serve as a potential biotechnology target for enhancing nitrogen efficiency in wheat and other crops.

## Supplementary Information

Supplementary Information 1.

Supplementary Information 2.

## Data Availability

The datasets generated in the current study are submitted in NCBI SRA database (PRJNA914888, PRJNA914883).
