# Supplementary material for: Long term nitrogen deficiency alters expression of miRNAs and alters nitrogen metabolism and root architecture in Indian dwarf wheat (Triticum sphaerococcum Perc.) genotypes
Source: Sci Rep. 2023 Mar 27;13:5002. doi: 10.1038/s41598-023-31278-4 (PMC10043004; doi:10.1038/s41598-023-31278-4)
Supplement: Supplementary file 1 — Supplementary Information 1. [file 41598_2023_31278_MOESM1_ESM.docx]

**
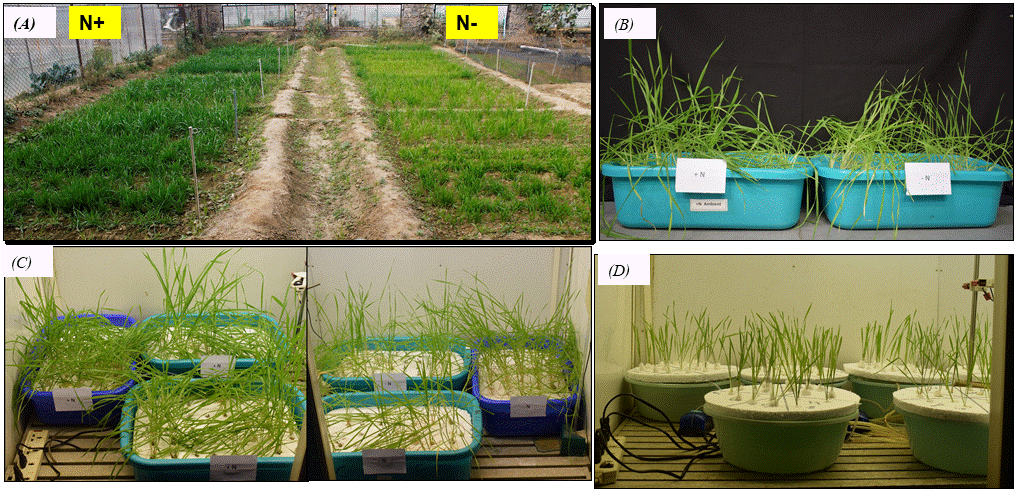
Supplementary Figure 1. Growth of wheat genotypes in field (A) rabi season (2018) with recommended N, 120 kg ha^-1^ (N+) and without N fertilizer application (N0) average soil N content before transplanting- 175 kg ha^-1^. (B), (C) Growth of wheat genotypes in Hydroponics N sufficient and (N+: 7.5 mM Nitrate), deficient (N-: 0.075 mM Nitrate) (D) Seedlings for miRNAseq study**

|   **(c)**  **(b)**  **(a)** |
| --- |
| **Supplementary Figure 2. Effect of nitrogen deficient (no applied N: N-) and nitrogen sufficient (120 kg ha^-1^ applied N: N+) field conditions on (a) leaf area, (b) vegetative shoot biomass accumulation and (c) total biomass accumulation of wheat genotypes** |

|   **(c)**  **(b)**  **(a)** |
| --- |
| Supplementary Figure 3. Effect of nitrogen deficient (no applied N: N-) and nitrogen sufficient (120 kg ha^-1^ applied N: N+) field conditions on (a) Plant Height, (b) Tiller No. plant^-1^ and (c) No. of Ears plant^-1^ of wheat genotypes. |

|   **(a)**  **(b)** |
| --- |
| **Supplementary Figure 4. Effect of nitrogen deficient (no applied N: N-) and nitrogen sufficient (120 kg ha^-1^ applied N: N+) field conditions on (a) No. of Spikelets Ear^-1^ and (b) Ear length of wheat genotypes.** |

**
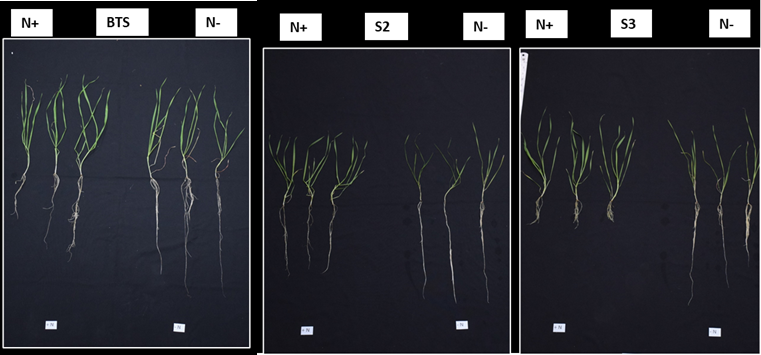
**

**D17**

**
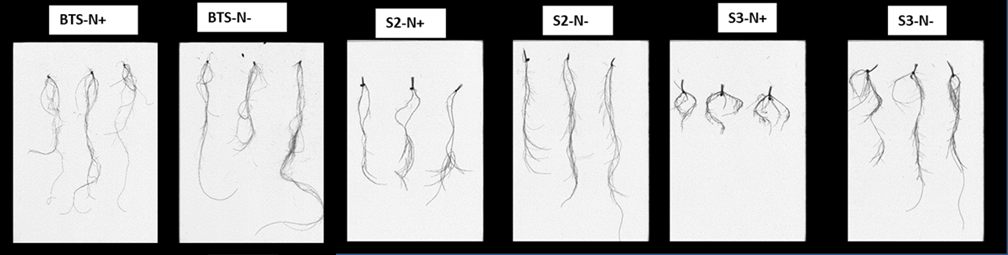
**

**Supplementary Figure 5. Comparison of plant and root growth of wheat genotypes in hydroponics receiving different nitrogen treatments; N+: 7.5 mM Nitrate, N-: 0.05 mM Nitrate.**
