# Supplementary material for: Long term nitrogen deficiency alters expression of miRNAs and alters nitrogen metabolism and root architecture in Indian dwarf wheat (Triticum sphaerococcum Perc.) genotypes
Source: Sci Rep. 2023 Mar 27;13:5002. doi: 10.1038/s41598-023-31278-4 (PMC10043004; doi:10.1038/s41598-023-31278-4)
Supplement: Supplementary file 2 — Supplementary Information 2. [file 41598_2023_31278_MOESM2_ESM.docx]

| **Code** | **Name of genotypes** | **Species** | **Other id** | **Country of Import to ICAR-NBPGR** |
| --- | --- | --- | --- | --- |
| S1 | EC 187182 | *Triticum sphaerococum* | KU- 161 | JAPAN |
| S2 | EC 0613055 | *Triticum sphaerococum* | 26793(SR 136V) | FRANCE |
| S3 | EC 187172 | *Triticum sphaerococum* | KU- 364 | JAPAN |
| S4 | EC 0613057 | *Triticum sphaerococum* | 26943 (SR.TUMIDUM PERCIV WRJ46453) | FRANCE |
| S5 | EC 576654 | *Triticum sphaerococum* | E-446 | UNKNOWN |
| S6 | IC 534882 | *Triticum sphaerococum* | PI-337997 | USA |
| S7 | EC 187181 | *Triticum sphaerococum* | KU- 9873 | JAPAN |
| S8 | EC 187183 | *Triticum sphaerococum* | KU- 162-1 | JAPAN |
| S9 | EC 180062 | *Triticum sphaerococum* | F11 | UNITED KINGDOM |
| S10 | EC 182945 | *Triticum sphaerococum* | CI 004923 | BRAZIL |
| S11 | EC 187167 | *Triticum sphaerococum* | KU- 304 | JAPAN |
| BTS | BT-SCHOMBURGK | *Triticum aestivum* | K-64386; AUS-25600 | Australia |

**Supplementary Table 1 Details of genotypes used in the study.**

|  | **Year1 N+ Vs Year2 N+** | | **Year1 N- Vs Year2 N-** | |
| --- | --- | --- | --- | --- |
|  | **Significant?** | **P value** | **Significant?** | **P value** |
| BTS | No | 0.216287 | No | 0.056417 |
| S1 | No | 0.028084 | No | 0.055374 |
| S2 | Yes | 0.002783 | No | 0.121714 |
| S3 | No | 0.028999 | No | 0.053009 |
| S4 | No | 0.033415 | No | 0.622243 |
| S5 | No | 0.018588 | No | 0.082283 |
| S6 | No | 0.504501 | No | 0.190848 |
| S7 | Yes | 0.003095 | No | 0.034779 |
| S8 | Yes | 0.000937 | No | 0.09735 |
| S9 | No | 0.42265 | No | 0.42265 |
| S10 | No | 0.648751 | No | 0.028133 |
| S11 | No | 0.400474 | No | 0.12638 |
| **Factors** | **C.D.** | **SE(d)** | **SE(m)** | **Significance** |
| Year (A) | 0.342 | 0.17 | 0.12 | * |
| N Level (B) | 0.342 | 0.17 | 0.12 | ** |
| Variety (C) | 0.837 | 0.416 | 0.294 | ** |

**Supplementary Table 2 Effect of nitrogen deficient (no applied N: N-) and nitrogen sufficient (120 kg ha^-1^ applied N: N+) field conditions on grain yield of wheat genotypes in 2018-19 and 2019-20.**
